# Gender-Specific Interactions Between Adiposity, Alcohol Consumption, and Biological Stress Biomarkers Among College Students in the United States

**DOI:** 10.3390/nu17162640

**Published:** 2025-08-14

**Authors:** Oladayo E. Apalowo, Meghan O’Dwyer, Edirisa J. Nsubuga, Leah Pylate, Abeer M. Alardawi, Nicole Reeder, Frank Kiyimba, Terezie Tolar-Peterson, Wes Schilling, Joel J. Komakech

**Affiliations:** 1 Department of Biochemistry, Nutrition and Health Promotion, Mississippi State University, Mississippi State, MS 39762, USA; mco133@msstate.edu (M.O.);; 2 Department of Food and Nutrition, King Abdulaziz University, Jeddah P.O. Box 80200, Saudi Arabia; aalardawi@kau.edu.sa; 3 Department of Nephrology, Vanderbilt University Medical Center, Nashville, TN 37232, USA; 4 Department of Health Science & Human Ecology, California State University, San Bernardino, CA 92407, USA; 5 Department of Family and Consumer Sciences, North Carolina Agricultural and Technical State University, Greensboro, NC 27411, USA

**Keywords:** obesity, alcohol, α-amylase, cortisol, stress, college students

## Abstract

Background: Obesity is a well-documented risk factor for cardiometabolic diseases associated with insulin resistance. However, research on its relationship with alcohol intake and stress markers, such as cortisol and α-amylase, remains limited, particularly among young adults in the general population. Objective: This study investigated the relationship between adiposity measures, alcohol intake, and biological stress biomarkers among college students. Methods: Participants (*n* = 189) completed the NIH Diet History Questionnaire II. Body composition was measured via bioelectrical impedance analysis. Salivary α-amylase (sAA) activity and cortisol (sCort) were assessed using the Salimetrics α-amylase kinetic enzyme assay and enzyme immunoassay kits, respectively. Multivariable linear regression models were used to determine the association between alcohol consumption and adiposity on biological stress biomarkers. Results: Among students who were overweight and obese, higher alcohol consumption increased sAA activity (β *=* 1.52, *p* = 0.030), with a greater effect in females (β = 2.24, *p* = 0.012). Body fat percentage showed similar patterns with sAA activity (β = 2.20, *p* = 0.015), with no significant effect in males. There was no significant interaction between BMI or body fat and alcohol consumption on sCort levels. However, significant main effects were observed for African Americans (β = 0.22, *p* = 0.020) and overweight and obese status (β = −0.19, *p* = 0.025) on male students’ sCort levels. African Americans (β = 0.21, *p* = 0.026) and young male adults within the underfat category (β = 0.35, *p* = 0.022) also exhibited increased sCort levels. Conclusion: Sex-specific patterns in physiological responses between males and females revealed stronger associations in females for sAA activity and distinct patterns in sCort levels among African American males.

## 1. Introduction

Elevated stress levels is a public health concern that is associated with poor mental health outcomes [1]. Stress-induced mental health disorders contribute to reduced quality-adjusted life years (QALYs), predisposing individuals to systemic illnesses and fatalities in extreme circumstances [2]. For university students, adjusting to college brings considerable academic and social adjustments, thus contributing to stress [3]. Social contextual factors contribute to chronic and acute stressors associated with people’s lifestyles and play a fundamental role in defining mental health risks [4,5,6]. Students face academic expectations and social anxiety, and when combined with chronic stress while attending a university, these conditions can lead to poor health outcomes and accelerate the risk of physical and mental health issues [7]. Weight gain and obesity are linked to recurrent episodes of excessive consumption of energy-dense foods and anxiety and anxiety-related behaviors in both young and older adults [8,9]. An analysis of NHANES 2015–16 data revealed a marked increase in obesity prevalence among emerging young adults in the United States, with 39.8% of individuals aged 20 and 25 classified as having obesity, and 7.6% being severely obese [10]. The high prevalence of people who are overweight, including obesity, continues to present significant public health challenges, more so in southern states such as Mississippi (40.1%), Alabama (39.2%), Tennessee (37.6%), and Louisiana (39.9%), and is also associated with all-cause mortality [11,12].

Obesity is a prevalent risk factor for various subtypes of cardiovascular disease (CVD) and cancers [13,14]. The economic cost of young adult obesity to healthcare systems is also expanding, altogether expected to reach USD 4.32 trillion annually by 2035, an increase from USD 1.96 trillion in 2020 if childhood overweight and obesity continue into adulthood, leading to increased lifetime expenses associated with obesity-related comorbidities and treatments [15,16,17]. Lifestyle choices, including nutrition, alcohol consumption, and physical activity, affect weight management in young adults beyond biological and genetic determinants [18]. Traditional nutritional assessment, which is solely reliant on body mass index (BMI) to identify overweight and obesity, is susceptible to misclassification. However, the Lancet Commission on Clinical Obesity recommends incorporating body fat anthropometric measurements like waist circumference and direct fat measurement in addition to BMI to minimize the risk of misdiagnosis of obesity [19].

Alcohol consumption has been associated with increased total energy consumption, potentially elevating the risk of becoming overweight and/or obese over time [20]. The health benefits of alcohol may be mediated by its effects on inflammation, platelet activation, lipid profile, fibrinogen, neurotransmitters, and social opportunities that alleviate social isolation [21], although the mechanisms remain obscure. The observed cardioprotective effects of alcohol may be due to residual confounding factors related to beneficial lifestyle, socioeconomic status, and behavioral characteristics with moderate alcohol consumption, yet not robustly examined in epidemiologic studies [22]. Lower-risk alcohol usage is alcohol consumption below a level thought to cause physical or psychosocial harm [23]. Unhealthy alcohol consumption includes any drinking that has physical, psychological, or social effects, which include at-risk drinking, severe episodic drinking (≥4 drinks (among women) or ≥5 drinks (for men) on a single occasion), and alcohol use disorder [23].

Biological stress regulation systems engage during stress and then return to the baseline afterward. However, chronic stress exposure can overwhelm the system and cause system failure, leading to inefficient regulation over time [24]. One of the principal components involved in the stress response is the hypothalamic–pituitary–adrenal (HPA) axis, which promotes cortisol synthesis in response to stress and establishes a pronounced diurnal pattern, with salivary cortisol (sCort) levels peaking in the early morning and declining throughout the evening [24]. There is evidence to suggest that chronically elevated cortisol levels have congruent effects. Cortisol enhances the consumption of sucrose and fat, induces insulin secretion, and redistributes energy stores and large abdominal depots [25,26]. This process generates an increased signal of abdominal energy stores, which inhibits catecholamines in brain centers that suppress cortisol activation and indicates how obesity may influence stress physiology [27]. The sympathetic nervous system (SNS) increases salivary alpha-amylase (sAA) levels by secreting norepinephrine [28]. sAA, an enzyme crucial for commencing starch digestion in the oral cavity, is encoded by the *AMY1* gene [29]. The copy number positively connects with sAA protein concentrations and enzymatic activity [30]. Moreover, sAA and sCort increase during chronic stress [31,32].

Due to the intricate relationship between alcohol consumption and obesity, it is crucial to investigate the synergistic impact of these factors on stress biomarkers, especially among young adults. This is necessary to enhance our comprehension of the physiological mechanisms underlying the relationship between regular alcohol consumption and obesity and facilitate early awareness and more precise dietary and public health recommendations. Due to the paucity of data concerning young adults and the absence of longitudinal data to elucidate potential causality, we investigated the interactive effects of alcohol consumption, adiposity, and salivary stress markers, sAA and sCort, among young adults attending college at a university in the Southern region of the US. Also, we examined the existence or nonexistence of sexual dimorphic patterns in salivary stress markers and participant adiposity described by BMI and body fat categories.

## 2. Methods

### 2.1. Study Sample and Procedures

**Figure 1:**
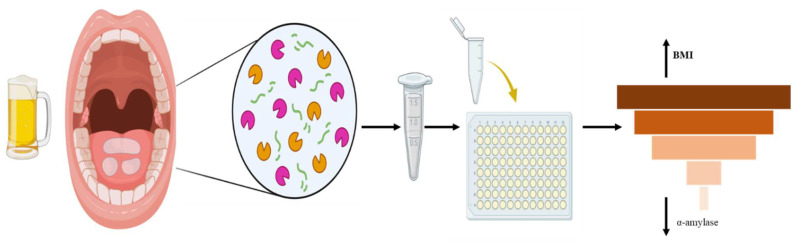
Schematic representation examining the complex relationship between alcohol consumption and salivary stress markers among college students in the United States.

**Figure 2:**
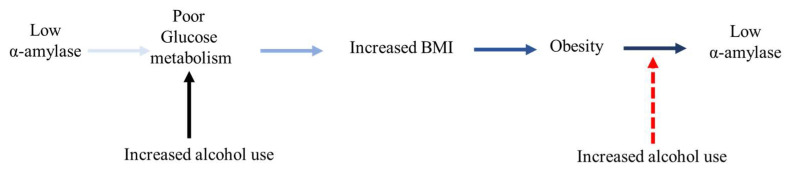
Bidirectional relationship between salivary α-amylase and obesity.

This study used previously collected data with study participants’ characteristics reported elsewhere under the BODY AP: Biological factors for Obesity Development in Young Adults Project study [33,34]. The current study, “Evaluating Dietary, Environmental, and Biological Factors for Obesity Development in Young Adults,” is a cross-sectional study investigating the influence of adiposity and alcohol consumption on biological stress markers: sAA and sCort. A schematic representation examining this complex relationship is provided in Figure 1 and Figure 2. The participants were students from a blinded university in the United States. The criteria for inclusion and exclusion in this study were 18 years of age or older, enrolled in college, not pregnant nor breastfeeding, capable of reading and writing in English, and willing to complete all study components, including providing a saliva sample. All participants were required to visit the university laboratory to conduct body composition measurements, submit a saliva sample, and complete associated surveys. During the one-time visit, the participants completed the web-based NIH Diet History Questionnaire II (DHQ II) and underwent body composition assessment using a bioelectrical impedance analysis machine. Additionally, adiposity was estimated using body mass index (BMI) for comparison. The original sample consisted of 217 young adults between 18 and 42 years old. After excluding 28 participants with missing information (e.g., alcohol intake, BMI, and saliva sample), the final analysis included 189 participants. This study received approval from the Institutional Biosafety Committee (IBC, IBC-25-039) and Institutional Review Board (IRB, IRB-24-622) from Blinded University. All procedures adhered to the ethical criteria set out by the IRB and the Helsinki Declaration of 1975, as amended in 2000. All participants provided informed consent before engaging in this study.

### 2.2. Diet History Questionnaire

The NIH web-based DHQ II food frequency questionnaire, which comprises 153 items, was administered to participants. The Nutrient Database and Diet*Calc software version 1.5.0 (https://epi.grants.cancer.gov/dhq2/, accessed from February 2016 to November 2020) were used to evaluate the data collected by the questionnaire instrument (DHQ) regarding food intake and portion sizes of the participants over the past 12 months.

### 2.3. Body Composition Assessment

A bioelectrical impedance analysis scale (MC-780, Tanita Corporation, Tokyo, Japan) was used to estimate body weight, total body fat percentage, and fat-free mass. The body fat percentage was determined by examining the correlation between body composition and fat content. The measures were implemented on Tanita equations that had been validated, with an emphasis on capacitance and induction. Additionally, for comparison, we also estimated the participants’ adiposity using the body mass index (BMI) based on the World Health Organization (WHO) classification [35]. Using the participants’ ratio of weight (kg) to height (m^2^), specific BMI categories included: underweight (BMI < 18.5), normal weight (BMI between 18.5 and 24.9), overweight (BMI between 25 and 29.9), and obese (BMI ≥ 30). BMI units were kg/m^2^.

### 2.4. Alcohol Intake Determination

The Alcohol Use Disorders Identification Test (AUDIT) is a 10-item questionnaire for alcohol screening, specifically developed to minimize cultural bias. The 10-item AUDIT questionnaire was used to gather data from participants regarding their drinking practices, alcohol consumption, and relevant alcohol-related issues. The research team extracted the initial three questions from ten questions called the AUDIT-Consumption (AUDIT-c) in this study [36]. AUDIT-c is a concise and efficient instrument for assessing alcohol intake, unlike the traditional 10-question assessment. The participants’ responses to the three alcohol consumption inquiries were analyzed. The AUDIT-c questionnaire yields total scores between 0 and 12, quantifying alcohol consumption. The categories of alcohol consumption encompassed beer, spirits, and wine. The standard serving size, frequency, and amount were documented; thereafter, alcohol consumption was computed by translating the reported quantity into grams per day (g/d).

### 2.5. Salivary Stress Biomarker Determination

#### 2.5.1. Sample Preparation

Saliva samples were completely thawed, vortexed, and centrifuged at 1500× *g* for 15 min to precipitate mucins and remove other particulate substances that could disrupt the test and influence the results. The samples were equilibrated to 20 °C before performing dilutions. A clear sample was then pipetted into appropriate dilution tubes for stress marker determination.

#### 2.5.2. sAA Assay

An α-amylase kinetic reaction assay kit (Catalog No. 1-1902-5, 5x96-Well Kit, LOT 2406537, Salimetrics LLC, State College, PA, USA) was used to test sAA. This kit is specifically designed and validated for the kinetic measurement of sAA activity and utilizes a chromagenic substrate, 2-chloro-p-nitrophenol, linked to maltotriose. Each assay contained 8 μL of controls (at high and low levels) and diluted saliva for sAA activity determination. The enzymatic action of α-amylase on this substrate was spectrophotometrically measured using a calibrated plate reader. The optical density (OD) at precisely 1 min was recorded, after which the plate was returned to mixing at 37 °C. The OD reading was retaken at exactly 3 min. The absorbance increases at 405 nm are directly proportional to the quantity of α-amylase activity in the sample. The results were reported in enzyme units per milliliter (U/mL) of α-amylase. The experiment was run across five assay kits. The plate means for high and low controls were calculated to determine the overall mean, standard deviation (SD), and % coefficient of variation (CV).

#### 2.5.3. sCort Assay

Cortisol was measured using an expanded-range high-sensitivity salivary cortisol enzyme immunoassay kit (Catalog No. 1-3002-5, 5x96-well plates, LOT 2408543, Salimetrics LLC, PA, USA) with a detection limit of 0.007 µg/dL. Each well plate contained 25 μL standard, control, and saliva samples. The cortisol in standards and samples competes with cortisol conjugated to horseradish peroxidase for antibody binding sites on a microtiter plate. Following incubation, unbound components were removed through washing. The bound cortisol–enzyme conjugate was quantified through the horseradish peroxidase reaction with the substrate tetramethylbenzidine (TMB), which produces a blue color. Upon terminating the reaction with an acidic solution, a yellow color was formed. The OD was measured using a conventional plate reader at 450 nm. The quantity of cortisol–enzyme conjugate was inversely proportional to the concentration of cortisol in the sample. Cortisol was reported as µg/dL.

### 2.6. Statistical Analysis

Descriptive statistics for selected participant characteristics were compiled. Multivariable linear regression models were utilized to analyze the association of alcohol consumption and adiposity on stress biomarkers: sAA and sCort. This study controlled for potential covariates, including participant age, ethnic group, and dietary factors such as total fat, protein, sugar, and dietary fiber. Continuous variables were mean-centered before analysis to enhance interpretability and mitigate potential multicollinearity. The regression coefficient (β) explained the interactive effects of our predictors on sAA and sCort. Exploratory analyses were performed to evaluate the multicollinearity of the explanatory variables. The variance inflation factor (VIF) (<10) and the tolerance test (<0.2) were within acceptable limits. Significance was set at *p* < 0.05. Data analyses were performed using STATA 18 (StataCorp LLC, College Station, TX, USA).

## 3. Results

### 3.1. Sociodemographic Characteristics of the Participants

The students’ mean age was 20 ± 2.6 years, with a bodyweight distribution of 38% overweight/obese, 54% normal weight, and 8% underweight. Students’ body fat distribution was 53% healthy, 30% overfat/obese, and 17% underfat (Table 1). Participants’ self-identified races were 65% Caucasian, 39% African American, and 3% categorized as other. The average daily dietary consumption for selected nutrients for the study participants is summarized in Table 2. Fiber intake was relatively low compared with that of other nutrients (16.7 ± 13.6 (g/d)), whereas total sugar consumption was the highest, with a 127.1 ± 111.2 (g/d) intake level among the listed nutrients. The corresponding macronutrient distribution in calories is as follows: total sugar, 508.4 kcal; total protein, 284 kcal; and total fat, 639 kcal, although with high variability. The average alcohol consumption was 9 ± 16.2 g/d, corresponding to 63 calories (1 g of alcohol contains 7 kcal [37]).

### 3.2. Salivary Stress Marker Characteristics

**Figure 3:**
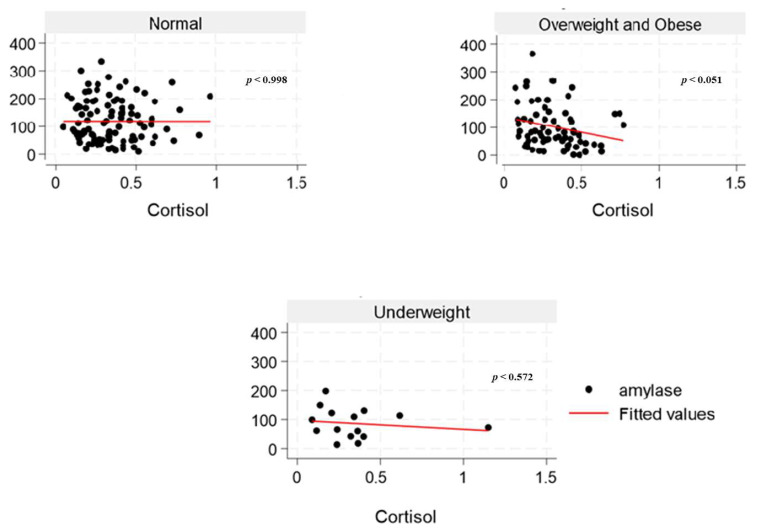
The relationship between salivary α-amylase (U/mL) and cortisol (µg/dL) levels across BMI categories (normal, overweight and obese, and underweight) among college students in the United States.

**Figure 4:**
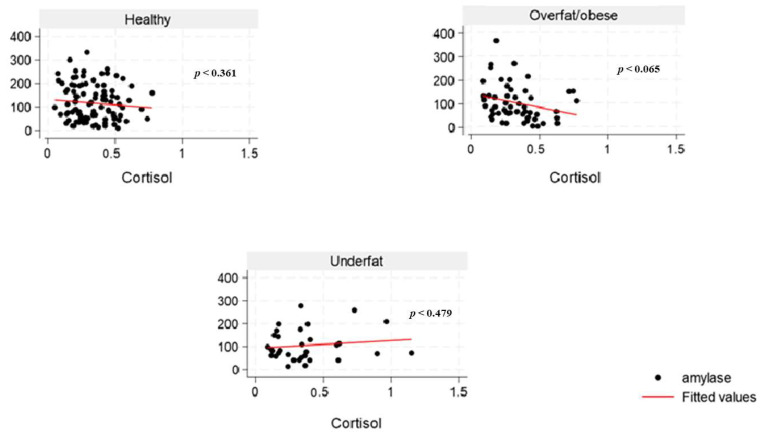
The relationship between salivary α-amylase (U/mL) and cortisol (µg/dL) levels across body fat percentage categories (healthy, overfat and obese, and underfat) among college students in the United States.

The inter-assay and intra-assay CVs for sAA were 18% and 3.4%, respectively. All samples did not exceed 400 U/mL (linearity limit at 1:200 dilution). The highest sAA activity was found to be 364.4 U/mL. However, two samples showed activity below 2.0 U/mL (0.98 U/mL and 1.97 U/mL), representing about 1% of the overall result. From our experiment, the inter-assay CV for sCort was 7.1%, and the intra-assay coefficient was 4.5%. The lowest and highest sCort levels obtained were 0.04 and 1.15 μg/dL, respectively, within the valid range. Our study findings showed a weak negative correlation between sAA activity and sCort levels across the overweight and obese (Figure 3) and the overfat/obese categories in the overall population (Figure 4), indicating an inverse relationship. Similarly, among the underfat cohort, a weak positive correlation was observed between amylase and cortisol, whereas this trend was reversed among the underweight cohort; however, these associations lack strength, potentially due to the significant heterogeneity/variation among the samples.

### 3.3. Interactive Effects of Alcohol Consumption and Participant Adiposity on sAA Activity

The results in Table 3 indicated that, within the final adjusted model, among students who were overweight and obese, each unit increase in alcohol consumption was associated with a 1.54-unit increase in sAA activity (*p* = 0.028). These findings implied increased stress levels for students in this category. However, sex-stratified analysis indicated that this relationship was most evident in females, where each unit increase in alcohol consumption was associated with a 2.26 unit increase in sAA activity (β = 2.26, R^2^ = 0.16, *p* = 0.012) in the overweight/obesity group, with no significant effects observed in males. Similar findings regarding body fat percentage were shown, as illustrated in Table 4. The sex-stratified analysis indicated that each unit increase in alcohol consumption was associated with increased sAA activity (β = 2.20, R^2^ = 0.15, *p* = 0.015) by more than double within the overfat/obese cohort; however, no significant effect was detected in males.

### 3.4. Interactive Effects of Alcohol Consumption and Participant Adiposity on sCort Levels

In this study, there were no significant interaction effects of BMI and alcohol consumption on sCort levels among all college participants combined (Table 5). Similarly, no combined effect of alcohol intake and body fat was observed in sCort levels for both female and male students combined (Table 6). Further, the omnibus models in Table 5 and Table 6 showed no significant main effects. Among male students only, significant main effects were observed for African Americans (β = 0.22, R^2^ = 0.43, *p* = 0.019) and overweight/obese status (β = −0.19, R^2^ = 0.43, *p* = 0.021) on sCort levels. Across body fat categories, whereas only a significant main effect was observed for African Americans (β = 0.21, R^2^ = 0.41, *p* = 0.026); young male adults within the underfat category were associated with an increase in sCort level (body fat; β = 0.35, R^2^ = 0.41, *p* = 0.022).

## 4. Discussion

This study provided key findings on the interactive effects of alcohol consumption and participant adiposity on the selected stress markers among college students. Initially, the results indicated that low sAA levels are associated with obesity. However, the higher alcohol use among young female adults with obesity significantly elevated sAA activity, suggesting a reversal in the trend observed in sAA activity in the absence of alcohol intake. Several studies have linked increased sAA activity with decreased odds of obesity [38], improved glycemic homeostasis [39], and an overall reduction in cardiometabolic risk [40]. Over the past decade, low serum amylase has been linked to metabolic syndrome (MetS), T2D, and non-alcoholic fatty liver disease (NAFLD), which is a hepatic manifestation of MetS and insulin resistance [41,42]. sAA influences individuals’ taste perception, thus modifying the satiety and appetite thresholds. Amylase expression in salivary glands is influenced mainly by genetic control. Numerous studies have indicated a substantial correlation between blood and sAA levels and the copy number variations (CNVs) of the sAA gene (AMY1) [43,44,45]. CNV of AMY1 exhibited an inverse correlation with BMI, insulin resistance, glucose tolerance, and predisposition to obesity. This suggests that high sAA activity may be associated with a rapid insulin response and a swift reduction in blood glucose levels following starch ingestion, which may protect against obesity via improved glucose metabolism and enhanced satiety.

Elevated amylase levels can be induced by carbohydrate-rich diets, which explains the observed positive correlation between total sugar and sAA and diminished sAA levels in young female adults with obesity in the current study. Low serum amylase levels in the overall population may be substantially influenced by obesity, which is associated with several cardiometabolic disorders, like impaired glucose tolerance and prediabetes, dyslipidemia, NAFLD, increased blood pressure, and insulin resistance [46]. In young women, excessive alcohol consumption, particularly when combined with high-fat dietary patterns and sedentary behavior, is associated with increased abdominal adiposity and overweight [47]. The pubertal transition in females marks a critical period of enhanced nutritional requirements for reproductive capacity. This developmental stage initiates several physiological adaptations characterized by the growth and maturation of multiple systems in the body, including musculoskeletal, neurodevelopmental, endocrine, metabolic, immune, cardiometabolic, and reproductive systems, with continued development extending into young adulthood [48]. An inadequate or suboptimal diet during adolescence and young adulthood may significantly impair reproductive fitness, leading to adverse health outcomes across generations [48]. Interestingly, studies indicate that cortisol levels frequently do not correlate with α-amylase under stress, implying that variations in α-amylase reflect a response to a stress signal that is independent of the limbic hypothalamic–pituitary–adrenal axis [49,50,51]. The inconsistent findings from the underweight (Figure 3) and underfat (Figure 4) cohort in the young male adult population regarding the correlation between sAA and sCort highlights the need to include body fat classification alongside BMI in obesity research. Moreover, while some studies have reported low sCort levels in individuals with obesity [52,53], there has not been any direct link between sCort across body fat categories.

Furthermore, a previous study examining human sAA activity determined that psychosocial stress increased sAA levels even in healthy individuals [54]. Some individuals use alcohol to alleviate stress, anxiety, and other negative emotions due to its short-term relaxation effects and effects on dopamine and stress reduction [55,56]. However, a study revealed that serum amylase level is associated with alcohol consumption, regardless of BMI [57]. This may result from injury to pancreatic tissue, such as chronic pancreatitis, and reduced sAA [58,59]. Nevertheless, the underlying mechanism may be complex, as the impact of alcohol on glucose homeostasis can vary depending on the quantity consumed [60], age, and overall lifestyle [61]. A study investigating sAA levels in connection with depressive symptoms indicates a propensity for elevated α-amylase levels in depressed individuals compared with non-depressed individuals; however, the findings are inconsistent [62]. α-Amylase hydrolyzes starch in the mouth and is autonomic and sensitive to stress-associated changes. The rapid autonomic nervous system (ANS) affects the circulatory and respiratory systems and releases catecholamines to help the body cope with stress [63].

Although alcohol consumption is known to modulate hypothalamic–pituitary–adrenal (HPA) axis activity, our study found no significant association between alcohol intake and salivary cortisol (sCort) levels, nor evidence of any interaction with adiposity across gender groups. The HPA axis regulates cortisol secretion and responds dynamically to both acute and chronic stressors. Acute alcohol exposure typically activates the HPA axis via corticotrophin-releasing factor (CRF), resulting in a transient increase in cortisol levels [64]. In contrast, chronic alcohol use is often associated with HPA hypo-responsiveness, likely driven by glucocorticoid receptor (GR) desensitization and negative feedback mechanisms [64,65,66]. Excess adiposity also influences HPA regulation through chronic low-grade inflammation and altered glucocorticoid receptor sensitivity [67,68], both of which can impair cortisol feedback signaling. Adipose tissue further contributes to neuroendocrine dysregulation by secreting pro-inflammatory cytokines such as interleukin-6 (IL-6) and tumor necrosis factor-alpha (TNF-α) [69], as well as adipokines including leptin and adiponectin [70], which may impair cortisol signaling and amplify stress reactivity. However, the absence of significant associations in our findings may reflect a blunted or desensitized HPA response, suggesting (1) chronic exposure among study participants, (2) alcohol and adiposity could exert independent rather than synergistic effects on cortisol regulation, and (3) it is plausible that any interaction is subtle and masked by unmeasured factors such as duration and pattern of alcohol use, history of stress exposure, or underlying metabolic phenotypes.

Furthermore, subgroup analyses revealed a distinct cortisol pattern in African Americans males, which may reflect population-specific vulnerability. These observations align with those in prior studies suggesting cortisol as a contributor to metabolic syndrome (MetS) pathogenesis among African Americans with obesity [71]. Cortisol is known to impair insulin sensitivity and contribute to hypertension, a condition that disproportionately affects young and middle-aged Black adults [72]. These metabolic disruptions increase the risk of serious health outcomes, including stroke, cardiovascular disease, and end-stage renal disease. Together, our findings align with those of broader biochemical models postulating that stress, alcohol use, and adiposity may converge on shared neuroendocrine pathways that influence metabolic health.

However, we observed significant interaction effects of alcohol consumption on salivary alpha-amylase (sAA) activity, particularly among young females with obesity (Table 3 and Table 5). This pattern suggests a sex-specific neuroendocrine response, potentially involving greater activation of the sympathetic–adrenal–medullary (SAM) axis, a key component of the autonomic nervous system. This finding aligns with that in prior work by Al Akl et al. [40], which reported strong female-specific associations between sAA levels, cardiovascular disease (CVD) risk markers, and proinflammatory cytokines in obesity. Elevated sAA may also indicate increased pancreatic stress, possibly aggravated by alcohol use, contributing to heightened amylase levels in this subgroup. Nevertheless, this requires further exploration, especially since rates of acute pancreatitis are comparable between genders, despite a higher prevalence of chronic pancreatitis in males.

Our study had some limitations, including the relatively small number of participants, which reduced the power analysis. Also, owing to the constraints of our study environment, the assessment of sCort and sAA among the students was not conducted at a standardized time post-awakening, which may inadvertently generate some inaccuracies in measurement. While salivary biomarkers have garnered significant attention due to their accessibility and ease of collection, the use of sAA has been identified as a sensitive biomarker for stress-induced physiological alterations indicative of sympathetic nervous system (SNS) activity, with an increasing number of studies substantiating the validity and reliability of this measure. However, sAA may not accurately reflect overall SNS activity. Another factor was that cardiac physiology and other indices of SNS activity pertinent to energy balance were not measured. The strengths of our study encompass a relative magnitude, largely diverse, high-risk sample featuring a significant proportion of people with obesity, classified by body fat and BMI and stratified by sex. While this study demonstrated a correlation between low sAA and obesity, the sample size for underweight and underfat was small, especially for subgroups of the male population. Another limitation was the possibility of recall bias among the participants’ dietary practices assessment.

Furthermore, since the current study used data from a previous study, we were limited to the confounding variables collected at the time. We recommend that future studies include additional variables, such as smoking status and physical activity, among others known to influence adiposity, which may strengthen the findings [73]. Despite these limitations, the participants constituted a homogeneous group regarding age and lifestyle, as they were all university students, which reduced variability and increased internal validity. Considering the sex differences identified in the present study, it is crucial to regard sex as a key moderator of these associations, even in larger populations and future research.

## 5. Conclusions

This study revealed significant sex-specific patterns in alcohol consumption and stress biomarker responses among college students who were overweight and obese. Different physiological responses were observed between males and females, with significant associations in females for sAA and distinct sCort patterns in males, particularly African Americans. Young college female adults with obesity had low sAA responses across body fat and BMI categories, but an opposing trend was observed due to alcohol consumption. Our novel findings suggest an association between adiposity and sAA activity and sCort levels. While these associations may be due to random variability based on individual responses, environmental factors, and other unpredictable factors, these physiological differences underscore the need for further investigation to develop strategies addressing dietary choices, including alcohol consumption practices in young adults of age, particularly females.

Although our study population consisted of college students, these findings establish a basis for future research. Future studies should examine the mechanisms by which alcohol consumption regulates sAA activity in obesity, including the potential mediation/modulation by additional socioeconomic, environmental, genetic, and hormonal factors. Epigenetic alterations and the transgenerational impact of alcohol consumption will facilitate the identification of viable therapies and adverse effects across generations. Subsequent studies should incorporate a larger sample categorized by age, adiposity, and sex. Understanding these physiological mechanisms could ultimately inform public health programs for more effective, targeted interventions that would reduce long-term health consequences related to interactions between adiposity, stress, and alcohol consumption during this formative life stage.

## Figures and Tables

**Table 1:** Participants’ demographics.

	Males and Females Combined	Females Only	Males Only
	*n* (%)	*n* (%)	*n* (%)
Variables	189	155 (82.01)	34 (17.9)
Age ^+^	20 ± 2.6	20 ± 2.7	21 ± 2.0
Ethnic Group			
Caucasian	122 (64.6)	105 (67.74)	17 (50)
African American	62 (32.8)	49 (31.61)	13 (38.24)
Others	5 (2.7)	1 (0.65)	4 (11.76)
BMI			
Normal	102 (53.97)	90 (58.06)	12 (35.29)
Overweight and Obese	72 (38.1)	50 (32.26)	22 (64.71)
Underweight	15 (7.9)	15 (79.68)	
Body Fat %			
Healthy	101 (53.44)	85 (54.84)	16 (47.06)
Overfat/obese	56 (29.63)	40 (25.81)	16 (47.06)
Underfat	32 (16.93)	30 (19.35)	2 (5.88)

^+^ Data are presented as the mean ± SD and *n* (%). BMI; body mass index, %; percentage. Body composition assessed using bioelectrical impedance analysis.

**Table 2:** Participants’ selected dietary intake per day.

Variables	Total ^+^
Alcohol consumption (g/d)	9 ± 16.2
Total fat (g/d)	71 ± 65.1
Total protein (g/d)	71 ± 70.5
Total sugars (g/d)	127.1 ± 111.4
Dietary fiber (g/d)	16.6 ± 13.6

^+^ Data are presented as mean ± SD. g/d: grams per day.

**Table 3:** Effects of alcohol consumption and BMI on salivary α-amylase levels among college students in the United States ‡.

	Males and Females Combined			Females Only			Males Only		
	Model-1	Model-2	Model-3	Model-1	Model-2	Model-3	Model-1	Model-2	Model-3
Variables	*n* = 189	*n* = 189	*n* = 189	*n* = 155	*n* = 155	*n* = 155	*n* = 34	*n* = 34	*n* = 34
Age	1.301	1.249	1.641	0.798	0.808	1.386	−0.201	0.053	0.885
Ethnic Group									
African American	8.641	11.551	8.575	12.090	16.198	12.331	−28.805	−23.469	−23.422
Others	−16.114	−14.260	−12.234	−118.193	−95.375	−97.061	−10.737	−1.092	−0.804
Diet									
Total Fat (g/d)	−0.157	−0.086	−0.006	−0.302	−0.235	−0.218	0.277	0.349	0.376
Total Protein (g/d)	0.038	−0.043	−0.105	0.220	0.248	0.216	−0.561	−0.641	−0.652
Total Fiber (g/d)	0.269	−0.043	−0.109	0.022	−0.767	−1.263	0.278	0.450	0.567
Total Sugar (g/d)	0.111	0.143 *	−0.162 *	0.150	0.178 *	0.220 *	0.1502	0.123	0.115
Alcohol Intake (g/d)		0.564	0.003		0.557	0.021		0.530	−0.583
BMI									
Overweight and Obese		−24.039 *	−24.772 *		−32.846 *	−32.279 *		10.884	11.520
Underweight		−30.342	−44.244		−30.725	−44.977			
BMI * Alcohol Intake									
Overweight and Obese			1.540 *			2.264 *			1.317
Underweight			−2.327			−2.257			
Pseudo R^2^	0.037	0.078	0.109	0.058	0.113	0.159	0.091	0.113	0.124
Δ Pseudo R^2^		0.04	0.03		0.05	0.05		0.02	0.01

Notes: All models are estimated with robust standard errors. * *p* < 0.05; ‡ values are β-coefficients; BMI—Body mass index (normal, overweight and obese, and underweight); Δ—change.

**Table 4:** Effects of alcohol consumption and body fat percentage on salivary α-amylase level among college students in the United States ‡.

	Males and Females			Females Only			Males Only		
	Model-1	Model-2	Model-3	Model-1	Model-2	Model-3	Model-1	Model-2	Model-3
Variables	*n* = 189	*n* = 189	*n* = 189	*n* = 155	*n* = 155	*n* = 155	*n* = 34	*n* = 34	*n* = 34
Age	1.301	1.499	1.781	0.798	0.971	1.229	−0.201	0.554	−2.405
Ethnic Group									
African American	8.641	15.668	14.848	12.090	20.933	19.039	−28.805	−29.978	−45.011
Others	−16.114	−15.038	−14.440	−118.193	−87.783	−86.710	−10.737	19.191	21.181
Diet									
Total Fat (g/d)	−0.157	−0.143	−0.090	−0.302	−0.304	−0.301	0.277	0.340	0.228
Total Protein (g/d)	0.038	0.047	−0.022	0.220	0.277	0.251	−0.561	−0.680	−0.608
Total Fiber (g/d)	0.269	−0.066	−0.178	0.022	−0.570	−1.051	0.278	0.616	0.713
Total Sugar (g/d)	0.112	0.137 *	0.152 *	0.150	0.173 *	0.208 *	0.151	0.136	0.153
Alcohol Intake (g/d)		0.648	0.201		0.681	−0.005		0.632	2.218
Body Fat									
Overfat/Obese		−28.268 *	−29.559 *		−39.642 *	−40.719 *		35.950	45.652
Underfat		−10.845	−11.382		−19.509	−19.704		97.521	−1096.895
Body Fat * Alcohol Intake									
Overfat/Obese			1.141			2.200 *			−2.330
Underfat			0.078			0.717			−139.900
Pseudo R^2^	0.037	0.075	0.088	0.058	0.117	0.153	0.091	0.220	0.295
Δ Pseudo R^2^		0.04	0.01		0.06	0.04		0.13	0.08

Notes: All models are estimated with robust standard errors. * *p* < 0.05; ‡ values are β-coefficients; body fat percentage assessed as healthy, overfat and obese, and underfat; Δ—change.

**Table 5:** Effects of alcohol consumption and BMI on salivary cortisol levels among college students in the United States ‡.

	Males and Females			Females Only			Males Only		
	Model-1	Model-2	Model-3	Model-1	Model-2	Model-3	Model-1	Model-2	Model-3
Variables	*n* = 189	*n* = 189	*n* = 189	*n* = 155	*n* = 155	*n* = 155	*n* = 34	*n* = 34	*n* = 34
Age	−0.001	−0.000	−0.002	−0.001	−0.001	−0.002	0.012	0.014	0.010
Ethnicity									
African American	−0.003	0.000	0.008	−0.032	−0.032	−0.024	0.198 *	0.223 *	0.223 *
Others	0.056	0.057	0.054	0.149	0.147	0.151	0.107	0.059	0.058
Diet									
Total Fat (g/d)	0.0002	0.000	0.000	−0.000	−0.000	−0.000	0.001	0.002	0.002
Total Protein (g/d)	−0.000	−0.000	0.000	0.001	0.001	0.001	−0.000	−0.001	−0.001
Total Fiber (g/d)	−0.001	−0.002	−0.001	−0.001	−0.001	−0.001	−0.000	0.001	−0.000
Total Sugar (g/d)	−0.000	−0.000	−0.000	−0.000	−0.000	−0.000	−0.001	−0.000	−0.000
Alcohol Intake (g/d)		0.000	0.001		0.000	0.001		0.003	0.009
BMI									
Overweight and Obese		−0.025	−0.023		0.002	0.001		−0.186 *	−0.190 *
Underweight		−0.007	0.052		0.001	0.058			
BMI * Alcohol Intake									
Overweight and Obese			−0.003			−0.003			−0.007
Underweight			0.012			0.012			
Pseudo R^2^	0.017	0.020	0.056	0.029	0.029	0.069	0.219	0.389	0.430
Δ Pseudo R^2^		0.00	0.04		0.00	0.04		0.17	0.04

Notes: All models are estimated with robust standard errors. * *p* < 0.05; ‡ values are β-coefficients; BMI—Body mass index (normal, overweight and obese, and underweight); Δ—change. Coefficients for diets (total fat, total protein, total dietary fiber, and total sugar) are too small to show any value.

**Table 6:** Effects of alcohol consumption and body fat percentage on salivary cortisol level among college students in the United States ‡.

	Males and Females			Females Only			Males Only		
	Model-1	Model-2	Model-3	Model-1	Model-2	Model-3	Model-1	Model-2	Model-3
Variables	*n* = 189	*n* = 189	*n* = 189	*n* = 155	*n* = 155	*n* = 155	*n* = 34	*n* = 34	*n* = 34
Age	−0.001	−0.001	−0.001	−0.001	−0.002	−0.002	0.011	0.021	0.015
Ethnicity									
African American	−0.003	0.001	−0.000	−0.032	−0.034	−0.034	0.198 *	0.214 *	0.151
Others	0.056	0.064	0.064	0.149	0.139	0.140	0.107	0.159	0.149
Diet									
Total Fat (g/d)	0.000	0.000	0.000	−0.000	−0.000	−0.000	0.001	0.001	0.001
Total Protein (g/d)	−0.000	0.000	−0.000	0.001	0.000	0.000	−0.000	−0.000	−0.001
Total Fiber (g/d)	−0.001	0.001	−0.001	−0.001	−0.001	−0.001	0.000	−0.001	−0.001
Total Sugar (g/d)	−0.000	0.000	−0.000	−0.000	−0.000	−0.000	−0.001	−0.000	−0.000
Alcohol Intake (g/d)		0.000	0.001		0.000	0.000		0.003	0.005
Body Fat									
Overfat/Obese		0.005	0.005		0.014	0.014		−0.037	−0.016
Underfat		0.039	0.036		0.012	0.011		0.354 *	−5.696
Body fat * Alcohol Intake									
Overfat/Obese			−0.001			−0.000			−0.003
Underfat			−0.002			−0.001			−0.701
Pseudo R^2^	0.017	0.023	0.027	0.029	0.031	0.031	0.219	0.418	0.507
Δ Pseudo R^2^		0.00	0.00		0.00	0.00		0.20	0.09

Notes: All models are estimated with robust standard errors. * *p* < 0.05; ‡ values are β-coefficients; body fat percentage assessed as healthy, overfat and obese, and underfat; Δ–change. Coefficients for diets (total fat, total protein, total dietary fiber, and total sugar) are too small to show any value.

## Data Availability

Data are available in the Supplementary Materials section.

## Supplementary Materials

The following supporting information can be downloaded at: https://www.mdpi.com/article/10.3390/nu17162640/s1, Table S1: Study participants’ characteristics.
